# *Chlamydia psittaci* pneumonia without cough: recovery, case report and literature review

**DOI:** 10.3389/fmed.2026.1764616

**Published:** 2026-03-27

**Authors:** Mingxuan Yang, Xue Yang, Ying He, Liqing Yao

**Affiliations:** 1 Department of Rehabilitation Medicine, The Second Affiliated Hospital of Kunming Medical University, Kunming, Yunnan, China; 2 Kunming Medical University, Kunming, Yunnan, China; 3 School of Rehabilitation, Kunming Medical University, Kunming, Yunnan, China

**Keywords:** case report, *Chlamydia psittaci*, pneumonia, pulmonary rehabilitation, unexplained fever

## Abstract

**Objectives:**

We report a case of *Chlamydia psittaci* pneumonia in a patient presenting with high fever and generalized myalgia but no cough. As infections with *Chlamydia psittaci* are potentially misdiagnosed and non-responsive to empirical antibiotic treatment, this report aims to enhance the awareness among the healthcare providers.

**Methods:**

A 34-year-old male patient developed high fever and fatigue after exposure to poultry. He presented to the hospital for treatment. Chest computed tomography (CT) showed a high-density shadow in the deep area of the left lower lung. Sputum culture returned negative. Empirical treatment with multiple antibiotics was given. No improvement was observed in the patient’s condition. Next-generation sequencing (NGS) was performed on bronchoalveolar lavage fluid. It confirmed the diagnosis of *Chlamydia psittaci* infection. The lesion was located deeply. The clinical team promptly adjusted the antibiotic regimen. Intensive pulmonary rehabilitation training was added simultaneously. It facilitated the drainage of deep-seated sputum. The patient finally achieved complete recovery and was discharged.

**Results:**

This case report indicates that in patients with high fever, clinicians should first determine the etiological factors and identify the lesion site. Accurate diagnosis can thus be achieved. In this case, the lesion was deeply located in the lung. Even with sensitive antibiotics, complete elimination of the lesion was difficult. Therefore, active pulmonary rehabilitation intervention should be implemented promptly. It promotes airway clearance, sputum excretion, and secretion drainage. It also accelerates the patient’s recovery process.

## Details of clinical case

**FIGURE 1:**
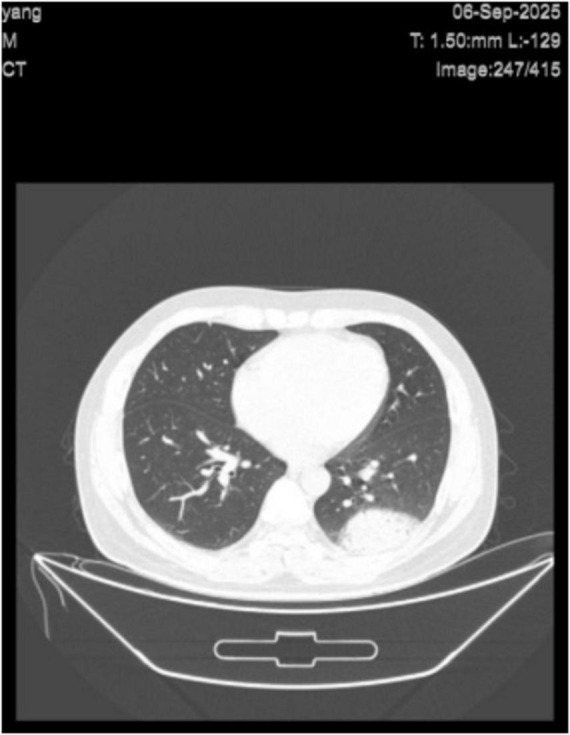
Axial cross-sectional CT scan of the chest clearly demonstrates the lungs, heart, and chest wall. A mass-like high-density opacity with surrounding haziness is observed in the left lower lobe, with adjacent costal pleural thickening, suggestive of infection. Minimal inflammation is also seen in the right lower lobe.

A 34-year-old male patient was admitted to the hospital due to high fever, generalized soreness, and weakness for 3 days. At the time of admission, the patient had a fever (39.6°/103.28°F) but had steady vital signs and did not display cough, nasal discharge, or chest pain. Physical examination findings were as follows: The patient displayed poor mental status with flushed face. His pharynx was slightly red and swollen, and thoracic mobility was normal. Auscultation of both lungs revealed no wheezes or rales. No other abnormalities were found in the remaining physical examination. The patient tested negative upon screening for respiratory pathogens, influenza A/B and COVID-19. Emergency routine blood test results showed: white blood cells (WBC): 6.48 × 10^9^/L, neutrophils: 5.36 × 10^9^/L, lymphocytes: 0.51 × 10^9^/L, monocytes: 0.60 × 10^9^/L, and eosinophils: 0.00 × 10^9^/L. Other inflammatory markers included: interleukin-6 (IL-6): 251 pg/ml, C-reactive protein (CRP): 189.72 mg/L, and procalcitonin (PCT): 0.29 ng/ml. Emergency chest computed tomography (CT) revealed: patchy high-density shadows with surrounding ground-glass opacity in the left lower lung lobe, and minimal ground-glass opacity in the right lower lung lobe (Figure 1). Based on clinical findings, pulmonary infection was considered. The patient was given ibuprofen suspension and compound aminopyrine barbital injection for antipyretic treatment. Empirical anti-infective treatment was sequentially initiated with ceftriaxone sodium, moxifloxacin, and piperacillin sodium and sulbactam sodium. However, all treatments showed poor efficacy, and the patient’s body temperature remained at 39.4 °C. The patient gradually developed sinus bradycardia, occasional ventricular premature beats, visual impairment, and drowsiness. Sputum culture tests were performed on 5 September and 8 September 2025. The patient could not expectorate sputum due to the deep location of the lesion. Both test results were negative. Blood culture tests were conducted three consecutive times on 5, 8, and 10 September 2025. All results were negative as well.

Given the patient’s prolonged fever of unknown origin, preoperative tests were completed. Bronchoalveolar lavage (BAL) was then performed. The lavage fluid was submitted for targeted next-generation sequencing (tNGS). Results came back one day later, confirming *Chlamydia psittaci* infection.

Further history-taking revealed that the patient had visited a pet exhibition half a month earlier. He had also spent some time in the parrot section.

After confirming the etiology, anti-infective treatment with doxycycline was initiated (Dosage and administration: 200 mg, intravenous drip, once daily). Active pulmonary rehabilitation was also started in combination. Meanwhile, ultrashort wave diathermy was applied for anti-inflammatory effects. Comprehensive chest clearance techniques were implemented as well.

First, postural drainage was given based on the lung lesion location. The patient was placed in lateral decubitus position. A soft pillow was put under the left chest to elevate it by 30–45°. The lower limbs were flexed and supported with pillows to ensure comfort. This procedure was done 2–3 times daily, 5–10 min each time. It allowed gravity to move deep-seated sputum toward the large airways. Second, chest percussion and vibration were performed. Percussion was delivered over the lesion-corresponding chest wall (left lower lung area) using a cupped hand or percussion hammer. The movement was directed from inferior to superior and from lateral to medial chest, at a frequency of 100–120 beats per minute. Force was adjusted according to the patient’s tolerance, to prevent chest pain or dyspnea. Right after percussion, vibration was applied: the palm was pressed firmly against the chest wall and vibrated rapidly (20–30 Hz) in time with the patient’s exhalation. This lasted for 3–5 respiratory cycles to improve sputum mobilization. In addition, the patient was taught proper coughing techniques: take a deep breath, hold it for 2–3 s, lean slightly forward, contract abdominal muscles forcefully, then cough to expel sputum by rapid thoracic contraction.

Moreover, high-frequency chest wall oscillation (HFCWO) was used to enhance deep sputum clearance. Initial parameters were set as follows: oscillation frequency 10–15 Hz (adult routine range: 5–25 Hz), pressure 3–5 kPa. Each treatment session lasted 10–15 min, twice daily. Frequency was gradually increased to 15–20 Hz and pressure to 5–7 kPa. Adjustments were made based on the patient’s tolerance (no chest pain or dyspnea) and sputum clearance effectiveness.

As doxycycline-induced liver damage was evident on the 6th day of treatment, it was discontinued and replaced with levofloxacin for anti-infective treatment (Dosage and administration: 500 mg, intravenous drip, once daily).

**FIGURE 2:**
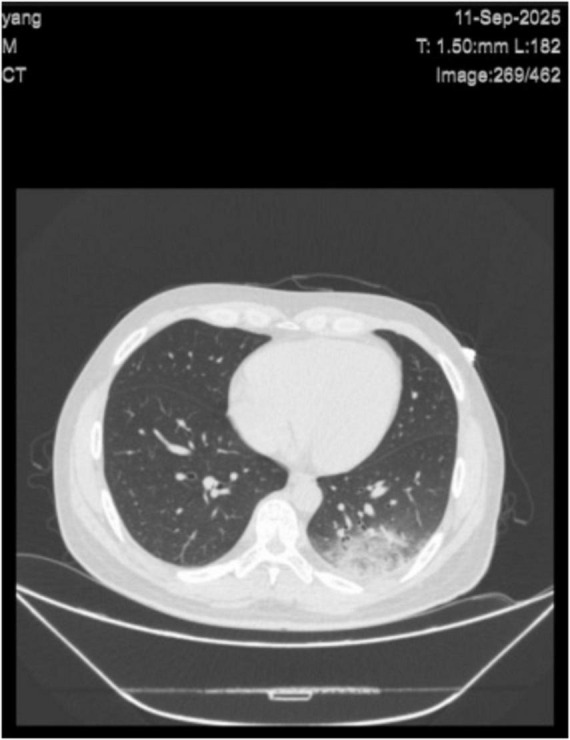
Axial cross-sectional CT scan of the chest shows the lungs, heart, ribs, and part of the spine with normal anatomical structures clearly visualized. Patchy haziness in the left lower lobe is decreased compared with prior images, indicating reduced infection. A tiny amount of left pleural effusion is noted.

Following the initiation of rehabilitation treatment, the patient developed a cough and expectorated a small amount of sputum, which was blood-tinged and viscous. Three days later, his body temperature gradually decreased and returned to normal, with no subsequent recurrence of fever. A follow-up chest CT showed patchy ground-glass opacities in the lower lobes of both lungs, suggestive of infection, which were more prominent in the left lower lung lobe (Figure 2). The patient’s mental status gradually returned to normal. A follow-up routine blood test showed no significant abnormalities, with inflammatory markers as follows: IL-6: 2.06 pg/ml, CRP: 17.45 mg/L, and PCT: 0.022 ng/ml. The patient requested discharge from the hospital. One month after discharge, he continued with rehabilitation training, and a follow-up CT scan showed no obvious lesions. The patient achieved full recovery. The detailed timeline is provided in Supplementary Appendix Table.

## Discussion

Psittacosis is a zoonotic disease caused by *Chlamydia psittaci*, an obligate intracellular, aerobic, Gram-negative bacterium (1). Its primary hosts are parrots and other avian species; humans acquire infection via contact with infected birds or their droppings, or inhaling C. psittaci-contaminated dust (2). There are no reports of such atypical cases to date. The occult presentation may increase the mortality rate of *Chlamydia psittaci* infection. It affects all age groups, predominantly adults (3), and mostly causes *Chlamydia psittaci* pneumonia—accounting for 1%–2% of community-acquired pneumonias (4).

Typical symptoms include high fever, cough, headache, myalgia, chills, and dyspnea. Untimely treatment can lead to severe pneumonia and death from multiple organ failure (5). Atypical cases (e.g., fever and myalgia without respiratory symptoms) are hard to diagnose due to difficult pathogen detection and non-specific blood tests (6). Given the high cost of metagenomic next-generation sequencing (mNGS), targeted next-generation sequencing (tNGS) with multiplex amplification is recommended as the first-line option for infections of unclear etiology. This technique offers the advantages of high sensitivity, rapid detection speed and low cost, but it cannot identify unknown pathogens or those not included in the targeted panel (7).

Doxycycline is the first-line treatment, with quinolones and macrolides as alternatives (8). Active pulmonary rehabilitation—such as ultrashort wave diathermy and airway clearance techniques (9–11)—aids pathogen excretion, enhances immunity, improves lung function, shortens hospital stays, and reduces costs. It has also benefited COVID-19 patients (12).

In this case, the patient showed a sharp increase in IL-6, CRP, and PCT, along with normal or decreased white blood cell counts, and low levels of lymphocytes, monocytes, and eosinophils. As the condition improves, various indicators gradually return to normal, suggesting that *Chlamydia psittaci* may impair the human immune function in the body (13, 14). Therefore, in cases where community-acquired pneumonia patients present with unexplained fever, show no significant response to empirical treatment, have severe conditions, and have suspected infections by other pathogens, mNGS testing should be performed immediately. This helps improve the diagnostic rate, allows timely adjustment of treatment regimens, reduces the use of broad-spectrum antibiotics, prevents further deterioration of the condition (15).

Regarding the three chest CT results of the case, they indicated that the pulmonary infection was concentrated in the left lower lobe of the lung, and the lesions gradually resolved as the condition improved. From the perspective of pathological changes, *Chlamydia psittaci* initially causes an inflammatory response around the local blood vessels in the lungs, leading to interstitial pneumonia. Ground-glass opacities around the centrilobular blood vessels can be observed in this stage (5, 16).

In the treatment after the cause was identified, the patient had no cough. This is attributed to the fact that the deep region of the left lower pulmonary lobe is distant from the bronchial tree, leading to relatively weak stimulation of the airway mucosa—an intensity insufficient to elicit the cough reflex. Furthermore, the segmental bronchi exhibit stenosis, which impedes the effective drainage of sputum. To achieve the goals of sputum excretion and inflammation resolution, timely and effective pulmonary rehabilitation intervention was administered in this case. Airway clearance techniques (ACTs) are one of the main components of respiratory rehabilitation for pulmonary infections. By improving collateral ventilation and interdependence, increasing expiratory flow velocity, and reducing the total airway cross-sectional ratio, ACTs enhance the generation of airway oscillations. Researchers have conducted large-scale prospective studies, which have demonstrated the effectiveness of ACTs in pulmonary rehabilitation, particularly during acute exacerbation phases (17). Commonly used ACTs include postural drainage with percussion, thoracic expansion exercises, forced expiration techniques, and high-frequency chest wall oscillation (HFCWO) or high-frequency chest wall compression (HFCWC). All the aforementioned techniques exert effects by increasing shear force, which in turn reduces the viscoelasticity of secretions and promotes the clearance of inflammatory secretions (Details of the specific treatment regimen are provided in the case section).

In addition, ultrashort wave diathermy (USWD) is a commonly used intervention in pulmonary rehabilitation. Characterized by deep penetration and low heat output, it is well suited for the acute phase of infection. The recommended dosage is non-thermal or mild thermal, with the power set at 40–60 W (suitable for deep lesions in the left lower lung). Non-thermal dosage helps alleviate inflammatory edema and prevent aggravated pulmonary congestion induced by heat generation. Each session lasts 10–15 min, once daily, with 5–7 days as one treatment course. Moreover, USWD has been demonstrated to alleviate inflammation and reduce lung injury by regulating macrophage polarization (11). Early administration of USWD in pneumonia may also stimulate and enhance the body’s resistance. In the case reported herein, timely implementation of the aforementioned pulmonary rehabilitation techniques yielded excellent therapeutic outcomes.

## Application to practice

A more in-depth understanding of *Chlamydia psittaci* pneumonia, together with heightened awareness of its atypical clinical manifestations, constitutes a fundamental prerequisite for improving patient outcomes. Prompt clinical diagnosis and targeted therapeutic interventions play a pivotal role in curbing disease progression and mitigating potential complications. Beyond conventional treatment strategies, the implementation of proactive, evidence-based pulmonary rehabilitation programs emerges as an indispensable component in facilitating the recovery process and restoring functional capacity among patients afflicted with this condition.

## Conclusion

This case emphasizes that *Chlamydia psittaci* pneumonia may present as high fever without cough, and highlights the importance of pulmonary rehabilitation intervention for the patient’s recovery. Accurate diagnosis, timely intervention, and sound rehabilitation strategies are crucial for the diagnosis and treatment of *Chlamydia psittaci* pneumonia.

## Data Availability

The original contributions presented in the study are included in this article/Supplementary material, further inquiries can be directed to the corresponding author.

## References

[1] Balsamo G Maxted AM Midla JW Murphy JM Wohrle R Edling TM et al. Compendium of measures to control *Chlamydia psittaci* infection among humans (Psittacosis) and pet birds (Avian Chlamydiosis), 2017. *J Avian Med Surg*. (2017) 31:262–82. 10.1647/217-265 28891690

[2] Zhang Z Zhou H Cao H Ji J Zhang R Li W et al. Human-to-human transmission of *Chlamydia psittaci* in China, 2020: an epidemiological and aetiological investigation. *Lancet Microbe*. (2022) 3:e512–20. 10.1016/S2666-5247(22)00064-7 35617977

[3] Vorimore F Thébault A Poisson S Cléva D Robineau J de Barbeyrac B et al. *Chlamydia psittaci* in ducks: a hidden health risk for poultry workers. *Pathog Dis*. (2015) 73:1–9. 10.1093/femspd/ftu016 25854003

[4] Xiao Q Shen W Zou Y Dong S Tan Y Zhang X et al. Sixteen cases of severe pneumonia caused by *Chlamydia psittaci* in South China investigated via metagenomic next-generation sequencing. *J Med Microbiol.* (2021) 70:1456. 10.1099/jmm.0.001456 34817316

[5] Yang N Ou Z Sun Q Pan J Wu J Xue C. *Chlamydia psittaci* pneumonia - evolutionary aspects on chest CT. *BMC Infect Dis*. (2025) 25:11. 10.1186/s12879-024-10374-4 39748281 PMC11697637

[6] Li H Hao B Wang Y Yu D Chen Z Du D et al. Metagenomic next-generation sequencing for the diagnosis of *Chlamydia psittaci* pneumonia. *Clin Respir J*. (2022) 16:513–21. 10.1111/crj.13519 35724965 PMC9329019

[7] Yin Y Zhu P Guo Y Li Y Chen H Liu J et al. Enhancing lower respiratory tract infection diagnosis: implementation and clinical assessment of multiplex PCR-based and hybrid capture-based targeted next-generation sequencing. *EBioMedicine*. (2024) 107:105307. 10.1016/j.ebiom.2024.105307 39226681 PMC11403251

[8] Kohlhoff SA Hammerschlag MR. Treatment of chlamydial infections: 2014update. *Expert Opin Pharmacother*. (2015) 16:205-12. 10.1517/14656566.2015.999041 25579069

[9] Volpe MS Guimarães FS Morais CC. Airway clearance techniques for mechanically ventilated patients: insights for optimization. *Respir Care*. (2020) 65:1174–88. 10.4187/respcare.07904 32712584

[10] McIlwaine M Bradley J Elborn JS Moran F. Personalising airway clearance in chronic lung disease. *Eur Respir Rev*. (2017) 26:160086. 10.1183/16000617.0086-2016 28223396 PMC9488523

[11] Li L Qu M Yang L Liu J Wang Q Zhong P et al. Effects of ultrashort wave therapy on inflammation and macrophage polarization after acute lung injury in rats. *Bioelectromagnetics*. (2021) 42:464–72. 10.1002/bem.22353 34130351

[12] Huang L Li Q Shah SZA Nasb M Ali I Chen B et al. Efficacy and safety of ultra-short wave diathermy on COVID-19 pneumonia: a pioneering study. *Front Med*. (2023) 10:1149250. 10.3389/fmed.2023.1149250 37342496 PMC10277738

[13] Chen J Sun Y Luo J Wu Y Wang K Zhang W et al. V-V ECMO for severe Chlamydia psittaci pneumonia presenting with sudden cardiac arrest: a case report and literature review. *Medicine*. (2024) 103:e39808. 10.1097/MD.0000000000039808 39533545 PMC11557096

[14] Fang C Xu L Lu J Xie Y Tan H Lin J. [Analysis of clinical features of 16 cases with *Chlamydia psittaci* pneumonia]. *Zhonghua Wei Zhong Bing Ji Jiu Yi Xue*. (2021) 33:1366–9. 10.3760/cma.j.cn121430-20210810-01159 34980310

[15] He L Yang H Liu S Liang W Zhou Z Long J et al. Physiological analysis of severe chlamydia psittaci pneumonia and clinical diagnosis after doxycycline-based treatment. *Front Physiol*. (2023) 14:1132724. 10.3389/fphys.2023.1132724 36846335 PMC9947341

[16] Radomski N Einenkel R Müller A Knittler MR. Chlamydia-host cell interaction not only from a bird’s eye view: some lessons from *Chlamydia psittaci*. *FEBS Lett*. (2016) 590:3920–40. 10.1002/1873-3468.12295 27397851

[17] Herrero-Cortina B Lee AL Oliveira A O’Neill B Jácome C Dal Corso S et al. European Respiratory Society statement on airway clearance techniques in adults with bronchiectasis. *Eur Respir J*. (2023) 62:2202053–90. 10.1183/13993003.02053-2022 37142337

