# Correction: *Chlamydia psittaci* pneumonia without cough: recovery, case report and literature review

**DOI:** 10.3389/fmed.2026.1857880

**Published:** 2026-04-29

**Authors:** Frontiers Production Office

**Affiliations:** Frontiers Media SA, Lausanne, Switzerland

**Keywords:** case report, *Chlamydia psittaci*, pneumonia, pulmonary rehabilitation, unexplained fever

The affiliation of reviewer Roshan Pais was erroneously given as “Rajiv Gandhi University of Health Sciences, India”. The correct affiliation is “CSI Lombard Memorial Hospital, Udupi, RGUHS, India”.

The original version of this article has been updated.

