# Supplementary material for: Chlamydia psittaci pneumonia without cough: recovery, case report and literature review
Source: Front Med (Lausanne). 2026 Mar 27;13:1764616. doi: 10.3389/fmed.2026.1764616 (PMC13065689; doi:10.3389/fmed.2026.1764616)
Supplement: Supplementary file 1 [file Data_Sheet_1.pdf]

Clinical Timeline of a Patient with Chlamydia psittaci Pneumonia

Date	Vital Signs	Laboratory Test Results	Antibacterial Treatment
2025.09.05	Body temperature 39.6℃; other vital signs stable; poor mental status; flushed face; mild pharyngeal swelling; no rales or wheezes auscultated in both lungs	1. Blood cell analysis: WBC 6.48$\times10^9$/L, neutrophils 82.6%, lymphocytes 7.9%, monocytes 0.60$\times10^9$/L, eosinophils 0.00$\times10^9$/L;<br>2. Mycoplasma pneumoniae antibody: Negative;<br>3. Sputum culture (first): Negative;<br>4. First blood culture: Negative	Initiated empirical anti-infective treatment with ceftriaxone sodium; simultaneous antipyretic treatment with ibuprofen suspension and compound aminopyrine barbital injection
2025.09.06	Body temperature maintained at 39.4℃; no cough, expectoration, nasal discharge, or chest pain	1. Blood cell analysis: WBC 6.08$\times10^9$/L, neutrophils 80.2%, lymphocytes 10.1%;<br>2. Inflammatory markers: IL-6 251 pg/ml, PCT 0.29 ng/ml, CRP 189.72 mg/L;<br>3. ESR 18 mm/h;<br>4. Respiratory pathogen test & COVID-19 test: Both negative;<br>5. Liver function: Albumin 37.9 g/L, ALT 42 U/L, AST 39 U/L;<br>6. Chest CT: Infection in the left lower lung lobe;<br>7. Abdominal CT: No abnormalities	Adjusted empirical anti-infective treatment to moxifloxacin
2025.09.07	Body temperature 39.3℃; gradually developed sinus bradycardia, occasional ventricular premature beats, visual impairment, and drowsiness	1. Blood cell analysis: WBC 5.79$\times10^9$/L, neutrophils 72.1%, lymphocytes 15.5%;<br>2. Inflammatory markers: IL-6 102.4 pg/ml, PCT 0.212 ng/ml, CRP 151.3 mg/L;<br>3. Sputum culture result (sampled on 2025.09.05): Negative	Adjusted empirical anti-infective treatment to piperacillin sodium and tazobactam sodium
2025.09.08	Body temperature 39.4℃; no cough; unable to expectorate sputum	1. Mycoplasma pneumoniae antibody: Positive;<br>2. CMV, EB virus, respiratory pathogen test, acid-fast smear, Widal test, TSPOT: All negative;<br>3. Sputum culture (second): Negative;<br>4. Second blood culture: Negative	Continued empirical anti-infective treatment with piperacillin sodium and tazobactam sodium
2025.09.10	Body temperature 39.2℃; no improvement in symptoms	1. Blood cell analysis: WBC 4.7$\times10^9$/L, neutrophils 56.6%, lymphocytes 27.7%, eosinophils 0.2%;<br>2. Sputum culture result (sampled on 2025.09.08): Negative;<br>3. Third blood culture: Negative;<br>4. Liver function: Albumin 33.4 g/L, ALT 394 U/L, AST 393 U/L (suggesting drug-induced liver injury)	Maintained piperacillin sodium and tazobactam sodium treatment; pending etiological confirmation for further adjustment
2025.09.11	Body temperature 39.4℃; no cough	1. Blood culture (2 aerobic + 2 anaerobic): No bacterial growth;<br>2. Bronchoalveolar lavage fluid (BALF) culture: No pathogenic bacteria or Haemophilus detected;<br>3. BALF targeted next-generation sequencing (tNGS) result: Confirmed Chlamydia psittaci infection	Discontinued piperacillin sodium and tazobactam sodium; initiated anti-infective treatment with doxycycline (200 mg, intravenous drip, once daily); combined with pulmonary rehabilitation (postural drainage, chest percussion and vibration, proper coughing techniques, high-frequency chest wall oscillation) and ultrashort wave diathermy
2025.09.12	Body temperature 39.4℃; cough developed after pulmonary rehabilitation; expectorated a small amount of blood-tinged [illegible]	Liver function: Albumin 35.8 g/L, ALT 278 U/L, AST 84 U/L, GGT 380 U/L (liver injury indicators improved compared to 2025.09.10)	Continued doxycycline anti-infective treatment + pulmonary rehabilitation

Date	Vital Signs	Laboratory Test Results	Antibacterial Treatment
2025.09.18	Body temperature returned to normal; no recurrence of fever; mental status recovered to normal	1. Blood cell analysis: No abnormalities;<br>2. Inflammatory markers: IL-6 2.06 pg/ml, PCT 0.022 ng/ml, CRP 17.45 mg/L (all close to normal levels);<br>3. Liver function: Albumin 35.3 g/L, ALT 261 U/L, AST 261 U/L, GGT 147 U/L;<br>4. Follow-up chest CT: Patchy ground-glass opacities in bilateral lower lung lobes (infectious manifestations), more prominent in the left lower lobe	Discontinued doxycycline due to drug-induced liver injury; switched to levofloxacin for anti-infective treatment (500 mg, intravenous drip, once daily); continued pulmonary rehabilitation
2025.09.24	Body temperature normal; no discomfort symptoms	Liver function: ALT 94 U/L, AST 35 U/L (liver injury indicators continued to improve)	Continued levofloxacin anti-infective treatment + pulmonary rehabilitation
1 month after discharge	No abnormal symptoms	Follow-up chest CT: No obvious lesions; all laboratory test indicators normal	Continued rehabilitation training after discharge; discontinued levofloxacin after completing the course
